# Influence of the Extraction Medium of Tannins from Eucalyptus Bark on the Properties of Rigid Tannin–Furfuryl Alcohol Foams

**DOI:** 10.1021/acsomega.5c10855

**Published:** 2026-02-11

**Authors:** Marlon Bender Bueno Rodrigues, Nayara Lunkes, Augusto Santos Do Nascimento, Fernanda Langone, Rodrigo Andrade Muraro, Otávio Schmalfuss Espíndola, Simone Pieniz, Darci Alberto Gatto

**Affiliations:** † Graduate Program in Materials Science and Engineering (PPGCEM), Technological Development Center, 37902 Federal University of Pelotas (UFPel), Pelotas 96010-610, Brazil; ‡ Materials Engineering Undergraduate Course, Technological Development Center, Federal University of Pelotas (UFPel), Pelotas 96010-610, Brazil; § Graduate Program in Food and Nutrition, Federal University of Pelotas, School of Nutrition, Pelotas 96010-610, Brazil

## Abstract

Eucalyptus bark is an abundant forestry residue in Brazil and a promising renewable source of condensed tannins for advanced material applications. Among them, tannin–furfuryl alcohol foams stand out as sustainable alternatives to petroleum-based foams due to their intrinsic fire resistance, low density, and high porosity. The performance of such foams is strongly influenced by the chemical profile of the tannins, which depends on the extraction medium. This study investigated tannin extraction from eucalyptus bark using distilled water, sodium hydroxide (NaOH), and sodium bisulfite (NaHSO_3_), and evaluated their impact on the structural, antioxidant, and functional properties of the extracts and on the performance of the resulting foams. Extraction yields were highest with NaOH (>50% for some clones), moderate with NaHSO_3_, and lowest with water. However, aqueous extracts showed superior antioxidant activity, whereas NaOH extracts displayed enhanced ferric-reducing capacity despite lower phenolic content. FTIR analyses confirmed solvent-dependent differences in hydroxyl, carbonyl, and aromatic groups. The foams produced exhibited distinct physical and mechanical behaviors. NaOH-derived foams showed the highest compressive strength (∼280 kPa) and smaller, denser cells, while NaHSO_3_-derived foams were lightweight and highly porous but structurally weaker. Aqueous extracts yielded intermediate strength but higher water uptake due to hydroxyl-rich compounds. All formulations maintained thermal stability above 400 °C and displayed self-extinguishing behavior under flame exposure. Nonetheless, incorporation of aqueous extracts slightly reduced fire resistance compared to control foams. These findings highlight the pivotal role of the extraction medium in determining tannin reactivity and, consequently, foam properties. Alkaline extraction favored mechanical performance, while aqueous extraction preserved phenolic-rich fractions with stronger antioxidant activity. This study links extraction chemistry directly to material performance. It contributes to the valorization of forestry residues into biobased foams, offering sustainable alternatives to synthetic polymeric materials.

## Graphical abstract

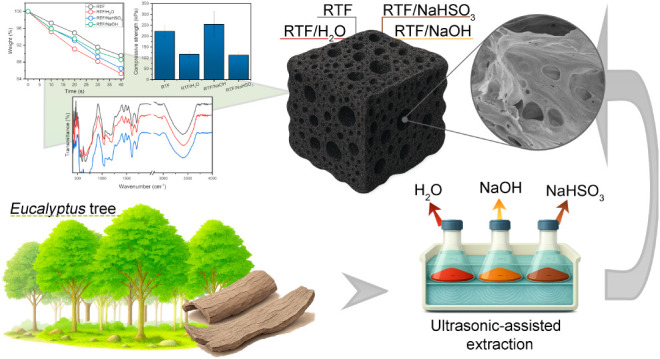

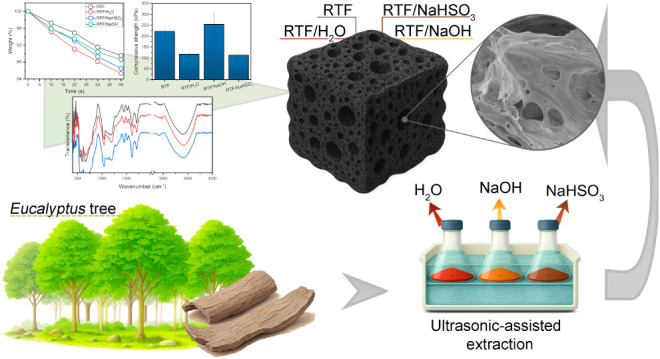

## 1 Introduction

Eucalyptus species are widely cultivated in Brazil, where they play a central role in the forestry industry due to their rapid growth, adaptability to different climatic conditions, and versatile applications. The country hosts one of the largest eucalyptus plantations in the world, primarily supplying the pulp and paper sector, but also providing raw material for energy production, wood-based panels, and chemical derivatives.^1^ In 2024, Brazilian eucalyptus planting covered 7.8 million hectares, corresponding to 76% of the total planted area—which represents a growth of 41% in the last ten years.^2^ Beyond their economic importance, eucalyptus trees are also a valuable source of bioactive compounds, among which tannins stand out for their chemical diversity and potential for high-value applications.^3,4^

Tannins are polyphenolic compounds naturally present in the bark, wood, leaves, and fruits of several plant species, where they act as defense agents against herbivores and pathogens. Chemically, they can be classified into hydrolyzable tannins, composed mainly of gallic or ellagic acid esters of glucose, and condensed tannins (or proanthocyanidins), which are oligomers or polymers of flavan-3-ols.^5^ The abundance of condensed tannins in eucalyptus bark makes this residue an attractive and sustainable source of polyphenols. Due to their high reactivity, tannins are already used in adhesives, wood panel resins, corrosion inhibitors, pharmaceuticals, and more recently in advanced materials such as foams, gels, and carbon-based structures.^6^ The extraction of tannins is a critical step that directly affects both the yield and the chemical profile of the recovered compounds. Tannin extraction from wood and bark involves separating polyphenols from polysaccharide matrices, often using inorganic or organic reagents.^7^ Various solvents can be employed, including aqueous acetone, ethanol, NaOH, and water, with each affecting the yield and quality of extracted tannins.^8^ For example, methanol has been found to be a more suitable solvent for tannin extraction compared to water, as demonstrated in a study on *Acacia xanthophloea* bark.^9^ Extraction methods typically involve alkaline solutions, with sodium hydroxide and sodium sulfite being common chemicals used. Water extractions, while yielding less, often produce better quality extracts.^10^ Alkaline solutions, such as NaOH, can promote the depolymerization of tannins and increase solubility, whereas bisulfite solutions (NaHSO_3_) stabilize reactive intermediates and lead to sulfonated derivatives with higher reactivity and water solubility.^11,12^ Thus, tailoring the extraction medium improves yield and modulates tannin properties, directly impacting their industrial potential.

Among these applications, thermosetting tannin–furfuryl alcohol foams have attracted significant attention as renewable alternatives to petroleum-based polymeric foams. These materials are obtained through the acid-catalyzed polycondensation of tannins with furfuryl alcohol, producing rigid, lightweight, and highly porous structures.^13^ Their properties, such as thermal stability, flame resistance, and mechanical strength, are strongly influenced by the structural characteristics of the tannins employed in synthesis. These foams exhibit comparable thermal insulation properties to synthetic counterparts while offering superior fire resistance and lower toxicity.^14^ Recent advances have focused on improving their physical and thermal properties. Tannin foams demonstrate excellent resistance to fire, chemicals, and solvents, with modifications using boric and phosphoric acids further enhancing fire resistance.^15^ While tannin foams show high water affinity and some inhomogeneity, they outperform polyurethane foams in thermal and fire resistance tests.^16^ Mechanical properties of tannin foams are comparable to phenolic foams, albeit with slight anisotropy and brittleness. Consequently, optimizing extraction methods is essential to ensure that tannins with the most suitable reactivity are obtained for foam production. By investigating different extraction media—such as water, NaOH, and NaHSO_3_—it is possible to understand how chemical treatments affect tannin structure and performance in advanced material applications.

This study focuses on the extraction of tannins from eucalyptus bark using distinct extraction media (water, NaOH, and NaHSO_3_) and evaluates how these treatments influence the chemical and functional properties of tannins, as well as their application in the production of rigid thermosetting foams. The results provide insights into the relationship between extraction chemistry, tannin reactivity, and the development of biobased materials, contributing to the valorization of forestry residues and the advancement of sustainable polymeric systems.

## 2 Materials and Methods

### 2.1 Raw Material

The raw material used in this study consisted of barks from four clones of forest species belonging to the genera *Eucalyptus*, kindly provided by CMPC Pulp and Paper. The clones were selected by the company according to pulp and paper yields and industrial interest. The chemicals required for the experiments were purchased as needed: sodium hydroxide (NaOH, CAS no. 1310-73-2), sodium bisulfite (NaHSO_3_, CAS no. 7631-90-5), formaldehyde (HCHO, CAS no. 50-00-0), diethyl ether (Et_2_O, CAS no. 60-29-7), sulfuric acid (H_2_SO_4_, CAS no. 7664-93-9), and furfuryl alcohol (C_5_HO_2_, CAS no. 98-00-0).

### 2.2 Extraction and Characterization of Tannins

The bark samples from 4 *Eucalyptus* clones were received in mid-March, separated, and stored in a climatic chamber. After stabilization in the climatic chamber, the samples were oven-dried at 105 °C until reaching constant weight and then ground in a Marconi MA340 knife mill (Piracicaba, Brazil). To obtain tannin-based liquid extracts, three solvents were used: distilled water, 0.5% NaOH solution, and 0.5% NaHSO_3_ solution.^17^ Using a 1:50 (g:mL) ratio, the extracts were prepared in beakers protected from ambient light and processed in a SolidSteel SSBuc ultrasonic bath (Piracicaba, Brazil) at 90 °C for 6 h. Two replicates (extractions) were carried out for each clone in each solvent. These extraction conditions (0.5% concentration, 90 °C, 6 h) were selected based on literature reports indicating that mild alkaline and bisulfite concentrations are sufficient to promote tannin solubilization while minimizing excessive degradation or polysaccharide coextraction. Although higher concentrations might increase yield, they often compromise tannin integrity. Regarding temperature,Amari et al. (2021) demonstrate that extraction at 90 °C maximizes yield for eucalyptus bark without inducing significant thermal degradation, which typically initiates above 150 °C for these tannins.^18^ Furthermore, the 6 h duration ensures sufficient mass transfer of condensed tannins from the bark, aligning with exhaustive extraction protocols for hardwood barks.^19^ Under these conditions, no evidence of severe thermal degradation was observed in preliminary screenings

### 2.3 Production of Condensed Tannin Foams

Rigid tannin foams were prepared as follows: 12 g of tannin (SETA natural polyphenol extract), 8 g of furfuryl alcohol (98%), 2.5 g of formaldehyde, and 2.4 g of distilled water (80 °C) were homogenized with a glass rod for 1 min. Subsequently, 2 g of diethyl ether (99.5%) were incorporated into the mixture, followed by manual stirring. Finally, 4.4 g of sulfuric acid (32%) were added to the solution, and the beaker was placed on a heated plate for curing. For the preparation of foams with clone-derived extracts, 10% of the total tannin content in the formulation was replaced by the produced extracts. For the preparation of foams with clone-derived extracts, 10% of the total tannin content in the formulation was replaced by the produced extracts. This specific proportion was established based on preliminary screening trials. It was determined that a 10% substitution was sufficient to introduce significant variations in the physical and mechanical properties of the foams—thereby proving the chemical activity of the extracts—without compromising the delicate kinetic balance between polymerization and blowing required for stable foam formation. Additionally, limiting the substitution to 10% aligns with resource efficiency principles by minimizing the consumption of purified extracts while still achieving functional modulation.

### 2.4 Analyses and Characterization

The antioxidant capacity of the extracts was evaluated using three radical-scavenging assays: FRAP (Ferric Reducing Antioxidant Power), DPPH (2,2-diphenyl-1-picrylhydrazyl), and ABTS (2,2′-azino-bis­(3-ethylbenzothiazoline-6-sulfonic acid)).

The FRAP assay was based on the reducing capacity of the extracts toward the ferric–triphenyltriazine complex (Fe^3+^–TPTZ). The reagent solution was prepared by mixing 10 mmol TPTZ in 40 mmol HCl, 20 mmol FeCl_3_·6H_2_O, and 300 mmol acetate buffer (pH 3.6), in a 1:1:10 (*v*:*v*:*v*) ratio. Subsequently, 3.0 mL of the freshly prepared FRAP solution was added to 0.1 mL of each sample. The reaction was carried out for 5 min under controlled conditions, and the color change—intensification of the blue hue—was monitored spectrophotometrically at 593 nm, being proportional to the antioxidant activity. Gallic acid (CAS no. 149-91-7) at a concentration of 0.55 mM was used as the standard, while a mixture of 3 mL of FRAP solution with 0.1 mL of distilled water served as the blank.

The free radical scavenging capacity against DPPH was evaluated using a stock solution of 60 μM DPPH in methanol. Under dark conditions, 0.1 mL of each extract was added to 3.9 mL of the DPPH solution in test tubes, followed by homogenization. A control solution was prepared by mixing 0.1 mL of a hydro-organic mixture (40 mL of 50% methanol, 40 mL of 70% acetone, and 20 mL of distilled water) with 3.9 mL of the DPPH solution. All samples were kept in the dark for 45 min before measurements. Absorbance was recorded on a spectrophotometer, with methanol as the blank. For calibration, DPPH solutions with concentrations ranging from 10 to 50 μM were used. Results were expressed as EC_50_ (μg·mL^–1^), corresponding to the concentration required to reduce 50% of the DPPH radical.

The ABTS•^+^ radical cation was generated by reacting ABTS solution (5.44 mM) with potassium persulfate (2.45 mM, final concentration), maintained in the dark for 16 h to allow formation of the stable radical. At the time of analysis, the solution was diluted with ethanol until an absorbance of 0.70 ± 0.02 at 734 nm was obtained. For the assay, 1 mL of the diluted ABTS•^+^ solution was placed in cuvettes, and absorbance was recorded before and after the addition of 10 μL of each extract, monitoring changes over 6 min. Ethanol was used as the blank. Results were expressed in μM Trolox equivalents per mL of extract.

The morphology of the produced foams was investigated using scanning electron microscopy (SEM) in both high- and low-vacuum modes, with a Jeol JSM 6610LV microscope (Akishima, Japan) operated at an accelerating voltage of 15 kV. ImageJ software (version 1.54f) was used to measure the average cell wall thickness, with more than 100 measurements taken per sample. The mean cell size was determined by Gaussian fitting of histograms.

FT-IR spectroscopy was performed using a Shimadzu Prestige-21 spectrometer (Kyoto, Japan) in attenuated total reflectance (ATR) mode. Spectra were collected from 600 to 4000 cm^–1^, with 32 scans per sample at a resolution of 4 cm^–1^.

Thermogravimetric analyses of the prepared extracts were conducted using a Shimadzu TGA-50 instrument (Kyoto, Japan). Mass loss curves were obtained over a temperature range of 30–600 °C at a heating rate of 10 °C·min^–1^ under an inert nitrogen (N_2_) atmosphere.

Fire resistance was evaluated according to the methodology of Tondi et al.^20^ Foam samples were cut into 2 × 2 × 2 cm^3^ cubes and exposed for 40 s to a flame (≥1200 °C) from a Bunsen burner. As tannin foams are inherently self-extinguishing, weight loss kinetics during flame exposure were monitored to compare and assess fire resistance among samples.

The compressive strength of the foams was determined using a TX-700 texture analyzer (Lamy Rheology Instruments, Lyon, France) equipped with a 50 N load cell. Following previous studies,^20^ compressive strength was defined as the stress corresponding to 20% deformation of the sample thickness, with a deformation rate of 0.10 mm·s^–1^.

The apparent density of the materials was calculated according to ASTM D1622—14. Specimens (5 × 5 × 2.3 cm) were weighed on a Shimadzu ATX analytical balance (Tokyo, Japan), with five replicates per formulation after stabilization at 25 °C and 65% relative humidity. The apparent porosity of the foams was calculated as the ratio between the apparent density of the materials and the bulk density of pellets produced in a hydraulic press under 2 tons of pressure for 2 min.

Five samples of each material were cut into 2 × 2 × 2 cm^3^ cubes and oven-dried at 60 °C until constant mass was reached to determine moisture content. The same samples were subsequently used to assess water absorption by immersing them in distilled water at 25 °C. At specific time intervals, specimens were removed, surface water was gently wiped off, and samples were weighed immediately.

## 3 Results and Discussion

The extraction yield represents one of the main criteria for assessing the technical and economic feasibility of obtaining plant extracts for application in materials such as rigid foams. Figure 1 shows the yield values (%) obtained for four sampled trees (A, B, C, and D), using the three different solvents.

**Figure 1:**
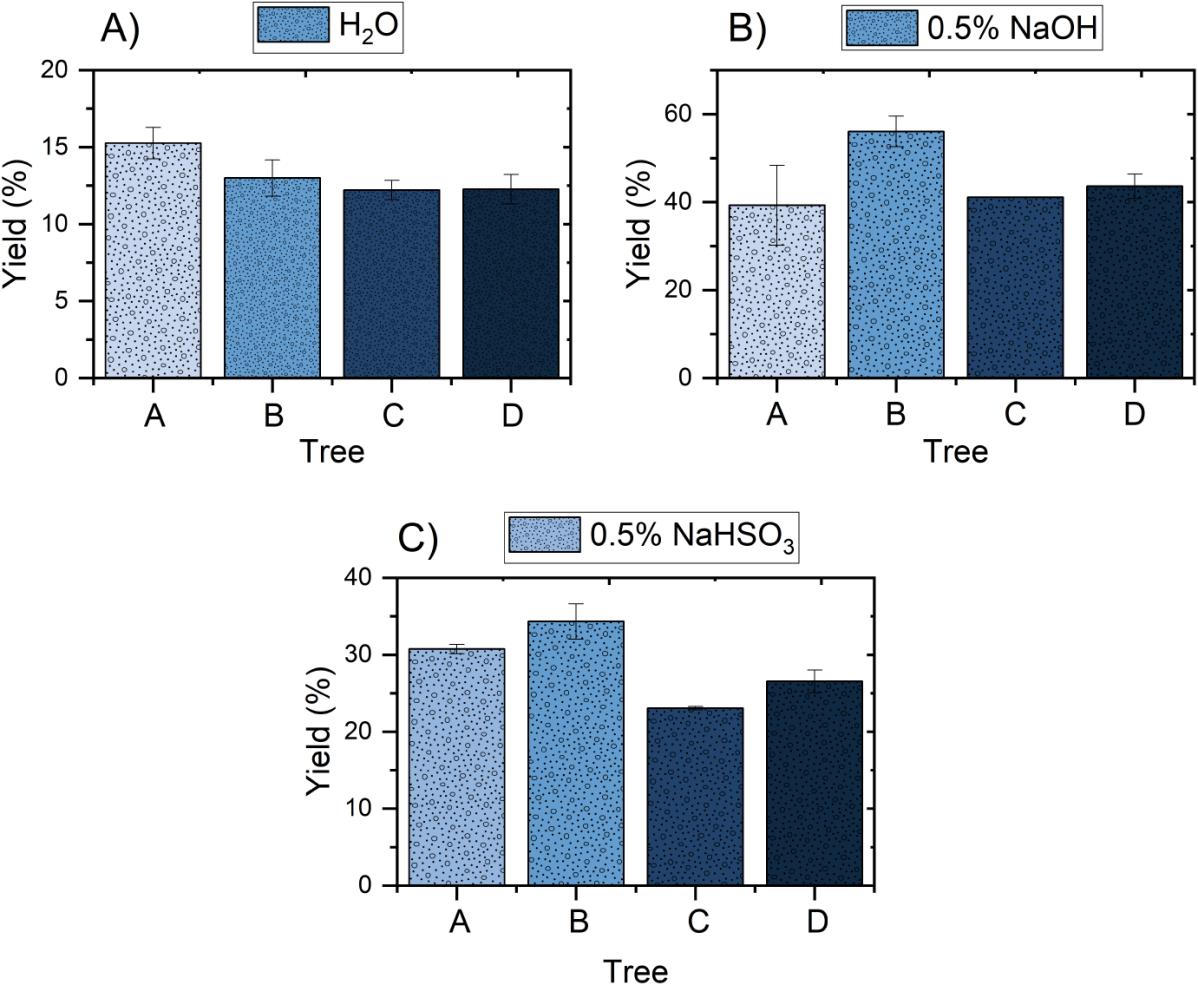
Extraction yield of eucalyptus clones using media containing (A) water, (B) 0.5% sodium hydroxide, and (C) 0.5% sodium bisulfite.

Both chemicals employed in this study, NaOH and NaHSO_3_, have their effectiveness in tannin extraction from tree bark reported in the literature, with applications in several tree species—e.g., chestnut, oak, spruce, and pine. Sodium hydroxide (0.5–1%) is particularly efficient in the extraction of hydrolyzable tannins, whereas sodium bisulfite (1%) shows better performance in the extraction of condensed tannins.^21,22^ Other compounds, such as sodium sulfite and sodium carbonate, can also enhance tannin extraction efficiency.^23,24^ It is important to note that extraction efficiency is influenced by factors such as temperature, extraction time, and solvent concentration, which were not selected for evaluation in the present study. Hot-water extraction at 140 °C yields high levels of total dissolved solids, although the tannin proportion decreases at temperatures above 100 °C.^23^ Similarly to the methodology proposed herein Quaratesi et al.^22^ performed ultrasound-assisted extraction using 0.5% NaOH at 50 °C for 10 min, which proved to be an energy-efficient method. For *Curupay* bark, a 3 h extraction with 3% sodium sulfite at 70 °C results in optimal tannin yield.

Among the tested solvents, 0.5% sodium hydroxide provided the highest extraction yields, reaching values above 50% in tree B, indicating greater efficiency in extracting soluble fractions. This efficiency is attributed to the alkaline cleavage of ester and ether bonds within lignin and hemicellulose structures. This mechanism facilitates the release of soluble compounds, including condensed tannins, structural sugars, and associated polyphenols.^25^

Sodium bisulfite also resulted in considerable yields, especially for tree B, which reached nearly 35%. The reducing and sulfonating action of NaHSO_3_ tends to increase the solubility of lignins and tannins through the introduction of sulfonate groups, thereby enhancing the extraction of compounds with higher affinity for polar solvents. However, compared to the alkaline medium, bisulfite extraction resulted in lower yields, which may be related to its lower efficiency in breaking down more recalcitrant cell wall structures.^26^ Water extracts exhibited the lowest yields, ranging from 12% to 16%, as expected, given water’s limited capacity to extract higher molecular weight or less water-soluble compounds. Aqueous extraction favors the solubilization of free flavonoids, soluble sugars, and other low-polarity secondary metabolites.

In addition to the solvent, the tree of origin also significantly influenced the extraction yields. Tree B showed the highest yields across all solvents, particularly in NaOH, suggesting greater structural susceptibility of its biomass to chemical action, possibly associated with lower density, higher porosity, or lower lignification degree. In contrast, trees C and D exhibited the lowest yields, especially in NaHSO_3_ and water extractions, which may reflect a less favorable composition for solubilization, such as higher condensed lignin content or lower amounts of free extractives.^27^ Tree A presented intermediate values under all conditions, behaving as a stable extraction matrix, although less efficient than tree B.

Despite the high yield provided by NaOH, it is important to highlight that extraction efficiency does not necessarily translate into higher phenolic content. As shown later, the *T*_NaOH_ extract presented a low total phenol content, indicating that the high yield may have been influenced by the extraction of less reactive or nonphenolic fractions, such as carbohydrates or humic substances. This dissociation between yield and functionality is critical for the rational selection of extracts to be applied in the formulation of materials such as tannin–furfuryl alcohol rigid foams.

The antioxidant capacity of the extracts obtained under different extraction conditions was evaluated using the FRAP, ABTS, and DPPH assays, in addition to the quantification of total phenolic compounds (TPC) by the Folin–Ciocalteu method (Figure 2).

**Figure 2:**
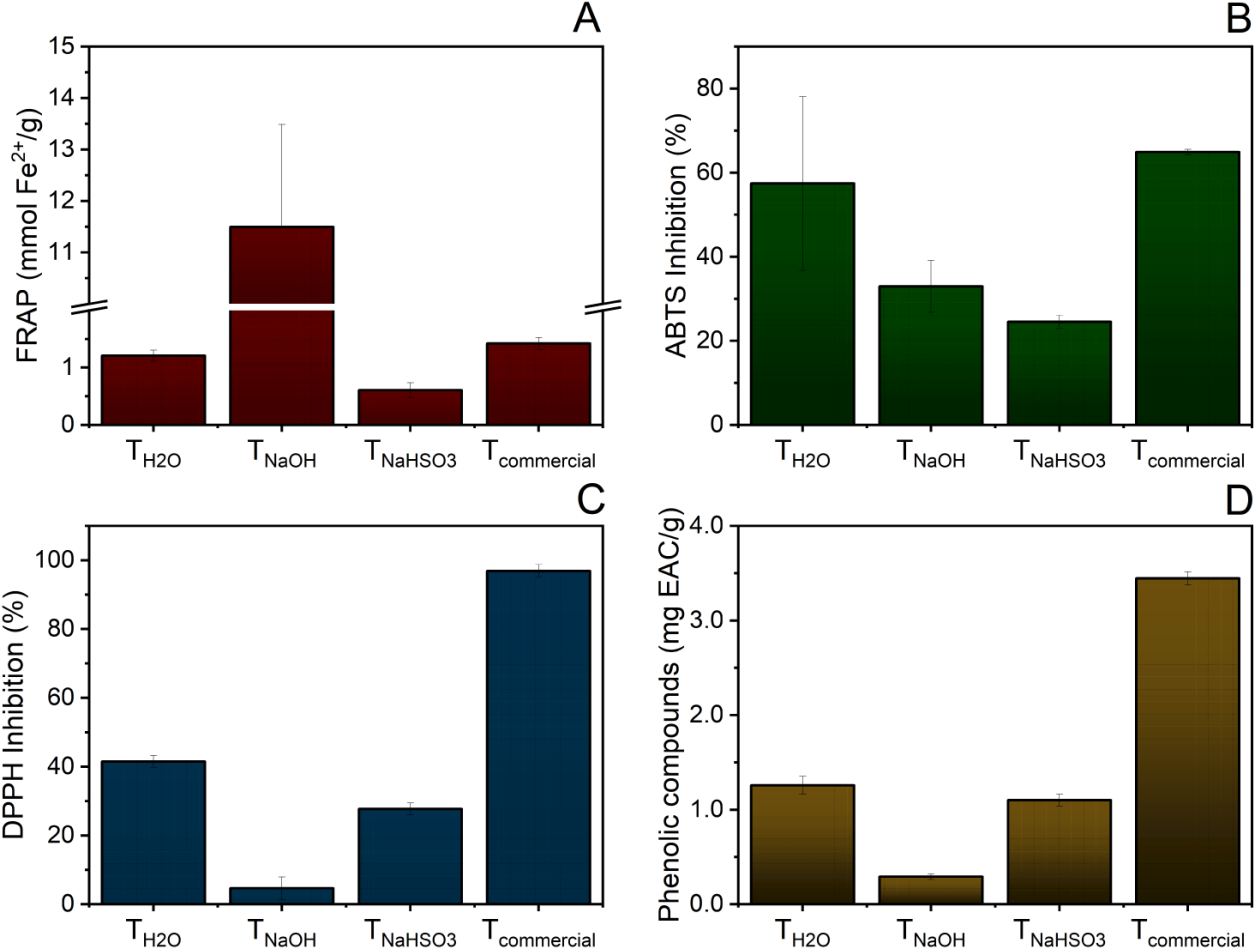
Active content analyses of the extracts produced by the methods (A) FRAP, (B) ABTS, (C) DPPH, and (D) phenolic compounds quantification.

The *T*_NaOH_ extract exhibited the highest FRAP value, reaching ∼12 mmol Fe^2+^/g, surpassing both the other extracts and the commercial tannin. This result suggests a higher content of compounds with strong reducing capacity, such as simple phenols or lower molecular weight tannins, whose redox activity is efficient in reducing ferric ions. Yue et al.^28^ investigated the extraction of polysaccharides from *Aspidopterys obcordata* Hemsl. using different solvents—hot water, HCl, NaOH, and 0.1 M NaCl—and found superior ferric ion reduction capacity in alkaline extracts compared with other solvents. Alkaline extraction likely promotes the cleavage of ester and ether bonds in the cell wall, releasing reactive phenolic structures.^29^

The *T*_H_2_O_ extract showed the highest ABTS radical scavenging activity among the natural extracts (∼60%), being surpassed only by the commercial tannin (∼65%). This result indicates the effectiveness of aqueous extraction in solubilizing antioxidant compounds with high reactivity against the ABTS^+^ radical. In contrast, the *T*_NaOH_ and *T*_NaHSO_3__ extracts exhibited lower activities, with inhibition below 40%, indicating that not all compounds extracted under these treatments display the required reactivity to neutralize anionic radicals. This behavior may be related to the polarity and solubility of the compounds obtained. Two in vitro studies addressed the relationship between water solubility and ABTS radical scavenging efficiency. In one study, a water-soluble curcumin complex (NDS27) showed increased ABTS scavenging efficiency, from 16% at lower concentrations to 46% at 10^–4^ M, while curcumin—dissolved in dimethyl sulfoxide due to its low water solubility—was qualitatively associated with inferior performance.^30^ In a second study, phenolic compounds (including Trolox, gallic acid, chlorogenic acid, and others) exhibited the highest electron transfer when dissolved in pH 7.4 buffer, while water resulted in the lowest reactivity.^31^ Highly condensed compounds, with lower solubility in aqueous solution, may display limited efficiency in the ABTS system, which relies on diffusion and interaction in aqueous medium.

The *T*_H_2_O_ extract also showed the highest activity among the natural extracts in the DPPH assay (∼42%), again ranking only below the commercial tannin, which reached nearly 100% inhibition. The DPPH radical is highly sensitive to hydrogen-donating groups such as simple phenols and flavonoids, whose extraction seems to be favored by aqueous solvent.^32^ However, some flavonoids, specifically certain dihydrochalcones and flavanones, may not react with DPPH, making the ABTS assay preferable for these compounds.^33^ In contrast, the *T*_NaOH_ and *T*_NaHSO3_ extracts exhibited poor performance (<30%), suggesting that although phenolic compounds were present, they were less efficient hydrogen donors or exhibited low solubility under the tested system.

The quantification of total phenolic compounds supported the results observed in the DPPH and ABTS assays. The commercial tannin showed the highest content (3.6 mg GAE/g), followed by the aqueous extract (∼1.3 mg GAE/g). The *T*_NaOH_ and *T*_NaHSO_3__ extracts presented significantly lower contents, despite the strong performance of *T*_NaOH_ in FRAP. This discrepancy highlights that the total phenolic content is not, by itself, a predictor of antioxidant capacity, as different classes of phenolic compounds exhibit distinct reactivities against the tested radicals. Studies examining the relationship between TPC and antioxidant capacity have yielded mixed results. While some research reported a positive correlation between TPC and antioxidant activity in tea infusions^34^ and common bean seeds,^35^ others described more complex relationships. Chaves et al.^36^ observed that the correlation between TPC and antioxidant activity in Mediterranean shrubs varied depending on the species group, extract concentration, and measurement method. They concluded that TPC alone is not a reliable indicator of antioxidant activity. In the case of *T*_NaOH_, the low total phenolic content may be compensated by the presence of structurally more efficient compounds in ferric ion reduction, although less effective in scavenging organic radicals. Table 1 summarizes the properties of the obtained extracts.

**Table 1:** Summary of the Physicochemical and Antioxidant Properties of Eucalyptus Bark Extracts Obtained Using Different Solvents

	*T*_H_2_O_	*T*_NaOH_	*T*_NaHSO_3__
Yield (%)	13.18	45.01	28.66
TPC (mg/EAC/g)	1.26	0.29	1.10
FRAP (mmol Fe^2+^/g)	1.21	11.49	0.61
ABTS inhibition (%)	57.44	32.94	24.55
DPPH inhibition (%)	41.53	4.64	27.69

Fourier-transform infrared (FTIR) spectroscopy (Figure 3A) enabled a qualitative assessment of the functional composition of the extracts obtained from biomass treated with different solvents. The comparison of spectra revealed significant variations in the intensity of bands associated with functional groups characteristic of phenolic compounds, carbohydrates, and carboxylic acids.

**Figure 3:**
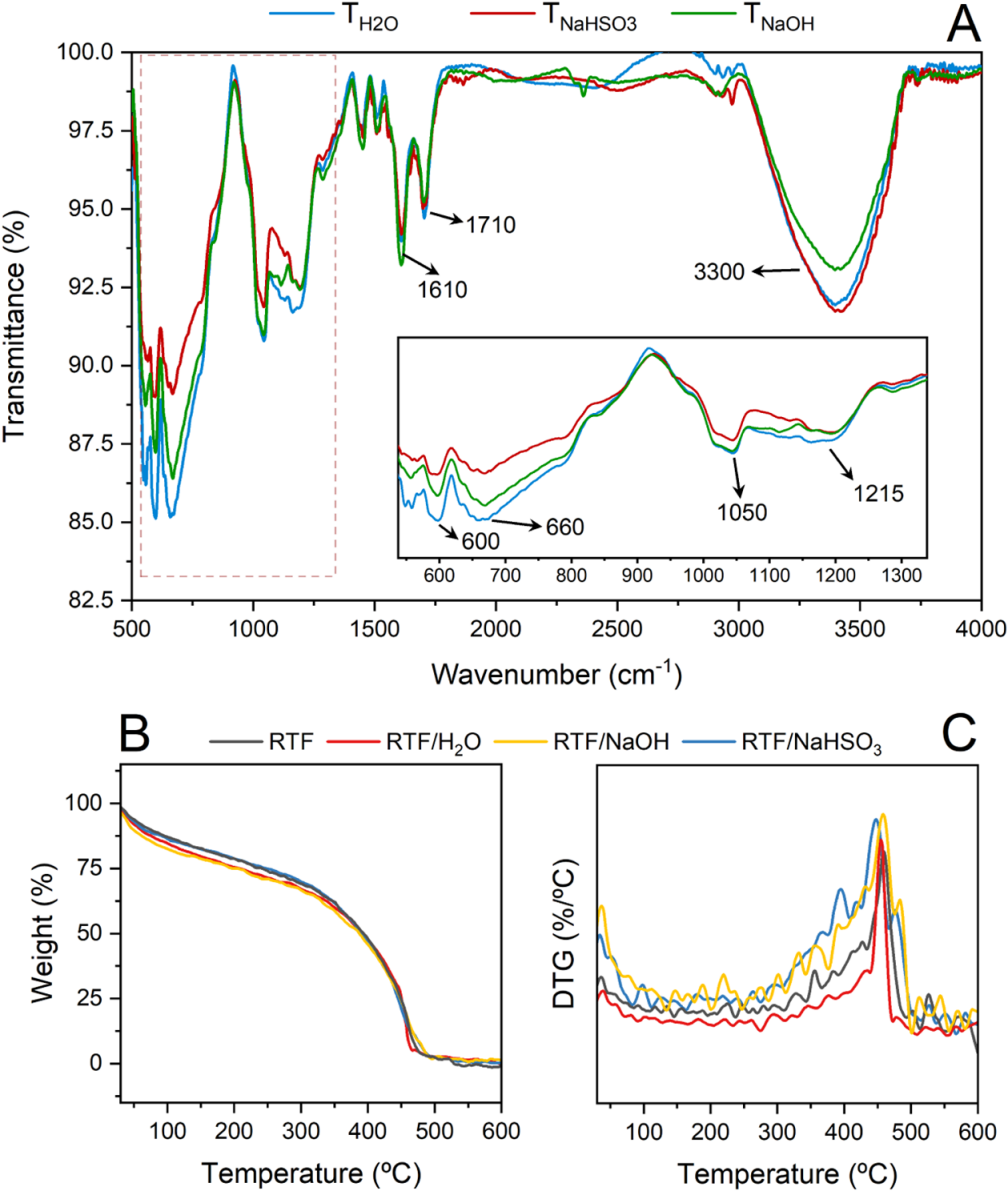
(A) Fourier-transform infrared spectroscopy (FTIR) curves of the produced extracts, (B) thermal degradation profiles, and (C) DTG curves of tannin–furfuryl alcohol rigid foams of the produced extracts.

The broad band centered at ∼3300 cm^–1^, attributed to O–H stretching vibrations, was observed in all extracts, with higher relative intensity in the *T*_H_2_O_ and *T*_NaHSO_3__ extracts. This band is associated with both phenolic groups and alcohols, as well as residual absorbed water, reflecting the abundance of hydroxyl groups. The lower intensity in *T*_NaOH_ suggests reduced solubilization of phenolic compounds and sugars with free hydroxyl groups, possibly derived from hydrolyzable or condensed tannins, whose extraction may not have been favored. According to Zhang et al.,^37^ alkaline-catalyzed treatments tend to preserve cellulose and hemicellulose—well-established methods in the literature—while degrading lignin and, consequently, other polyphenolic agents. Similarly, Canbolat, Ozkan & Kamalak^38^ reported that NaOH treatment linearly decreased (*p* < 0.001) the condensed tannin content in extracts obtained from *Arbutus andrachne* and *Glycyrrhiza glabra* leaves.

The band at ∼1710 cm^–1^, typical of C=O stretching in carboxylic acids or esters, was most prominent in the aqueous extract, indicating the possible extraction of oxidized compounds such as phenolic acids or oxidative degradation products of lignin.^39^ Its reduced intensity in *T*_NaOH_ and *T*_NaHSO_3__ may be attributed to carbonyl hydrolysis or saponification of esters under alkaline conditions.^40^ The band near 1610 cm^–1^ was assigned to C=C stretching vibrations in aromatic rings, typical of lignin and condensed tannins.^41^ This band was more intense in *T*_NaOH_ and *T*_NaHSO_3__, indicating greater extraction of aromatic substances under the action of these solvents. In particular, bisulfite acts as a reducing agent, promoting lignin bond cleavage and enhancing the extraction of sulfonated phenolic fractions.

The bands at 1215 cm^–1^ and 1050 cm^–1^ correspond to C–O and C–O–C stretching vibrations, associated with carbohydrates including cellulose, hemicelluloses, and phenolic glycosides. Their higher intensity in *T*_H_2_O_ suggests that the aqueous medium favored the extraction of polyols, mono- and oligosaccharides, by cleaving hemicellulosic linkages.^42^ The 1050 cm^–1^ band is particularly associated with xylan and mannan fractions.^43^ The bands in the 660–600 cm^–1^ region, although less intense, are related to out-of-plane vibrations of substituted aromatic rings, common in condensed tannin structures. Their varying intensities suggest different proportions of aromatic structures extracted depending on the solvent, reinforcing the selectivity of each treatment.

These structural differences identified by FTIR are pivotal for understanding the subsequent foam performance. For instance, the high intensity of bands associated with hydroxyls (∼3300 cm^–1^) and glycosidic linkages (1050 cm^–1^) in the *T*_H2O_ extract indicates a significant presence of soluble sugars and polyols. These compounds act as plasticizers within the rigid tannin-furanic network, similar to the effect of glycerol observed in flexible tannin foams.^44^ This plasticizing effect interferes with the rigidity of the matrix, resulting in the lower compressive strength and higher water uptake observed for RTF/H_2_O foams. Conversely, the removal of these nontannin hydrophilic fractions typically enhances mechanical performance, as demonstrated by C̆op et al.,^45^ supporting our observation that the *T*_NaOH_ extract—which favored aromatic fractions over sugars—yielded a mechanically superior cellular matrix. At a molecular level, the cleavage of lignin-carbohydrate complexes by NaOH likely increases the availability of reactive phenoxide ions. These species are highly nucleophilic and accelerate the polycondensation with furfuryl alcohol, resulting in a tighter cross-linked network compared to the water-extracted tannins, where steric hindrance from solvated sugars impedes efficient polymerization.

Thermogravimetric analysis (TGA) of the foams (Figure 3B) revealed degradation profiles typical of polymeric materials with aromatic character, showing a continuous and progressive mass loss from room temperature up to ∼450 °C. This gradual decomposition is associated with the complex polymeric network formed between tannins and furfuryl alcohol, whose cross-linking products are highly aromatic and partially carbonizable, thus conferring greater thermal stability compared to conventional polymers. The DTG curve (Figure 3C) shows that all foams exhibit a main degradation event between 400 and 500 °C, with variations in peak intensity and temperature depending on the extract employed. This event is attributed to the degradation of the furanic–phenolic polymeric backbone, which constitutes the continuous phase of the foam.^46^ The RTF/NaHSO_3_ foam displayed the most intense DTG peak, shifted to the lowest temperature of the group, suggesting that the presence of functionalized groups from saline extraction may have promoted structural rearrangements in the network, possibly leading to domains of lower thermal stability. In contrast, the RTF/NaOH and RTF/H_2_O foams exhibited DTG peaks slightly shifted toward higher temperatures, which may indicate a more homogeneous structure or a slightly higher degree of cross-linking, thereby enhancing thermal resistance.

The observed differences can be correlated with the FTIR results, since alkaline extracts showed more pronounced bands in the 1215–1050 cm^–1^ region, assigned to C–O and C–O–C linkages, which may participate in cross-linking with furfural but can also introduce thermal instabilities when present in excess.^47^ Moreover, the lower total phenolic content in alkaline extracts may have negatively affected the cross-link density of the foams, which is consistent with the higher degradation rate observed in DTG. Although all foams display a similar thermal behavior, with major decomposition occurring above 400 °C, the type of extract influences both the intensity and profile of degradation, reflecting the complex interplay between chemical composition, cross-linking density, and the thermal stability of the tannin–furfuryl polymeric matrix.

The physical properties of the foams, such as density, porosity, and compressive strength, are directly related to the morphology of the cellular structure formed during polymerization and to the type of additive incorporated. Figure 4 presents the results of bulk density (A), porosity (B), compressive stress (C), and specific stress (D) for the different formulations.

**Figure 4:**
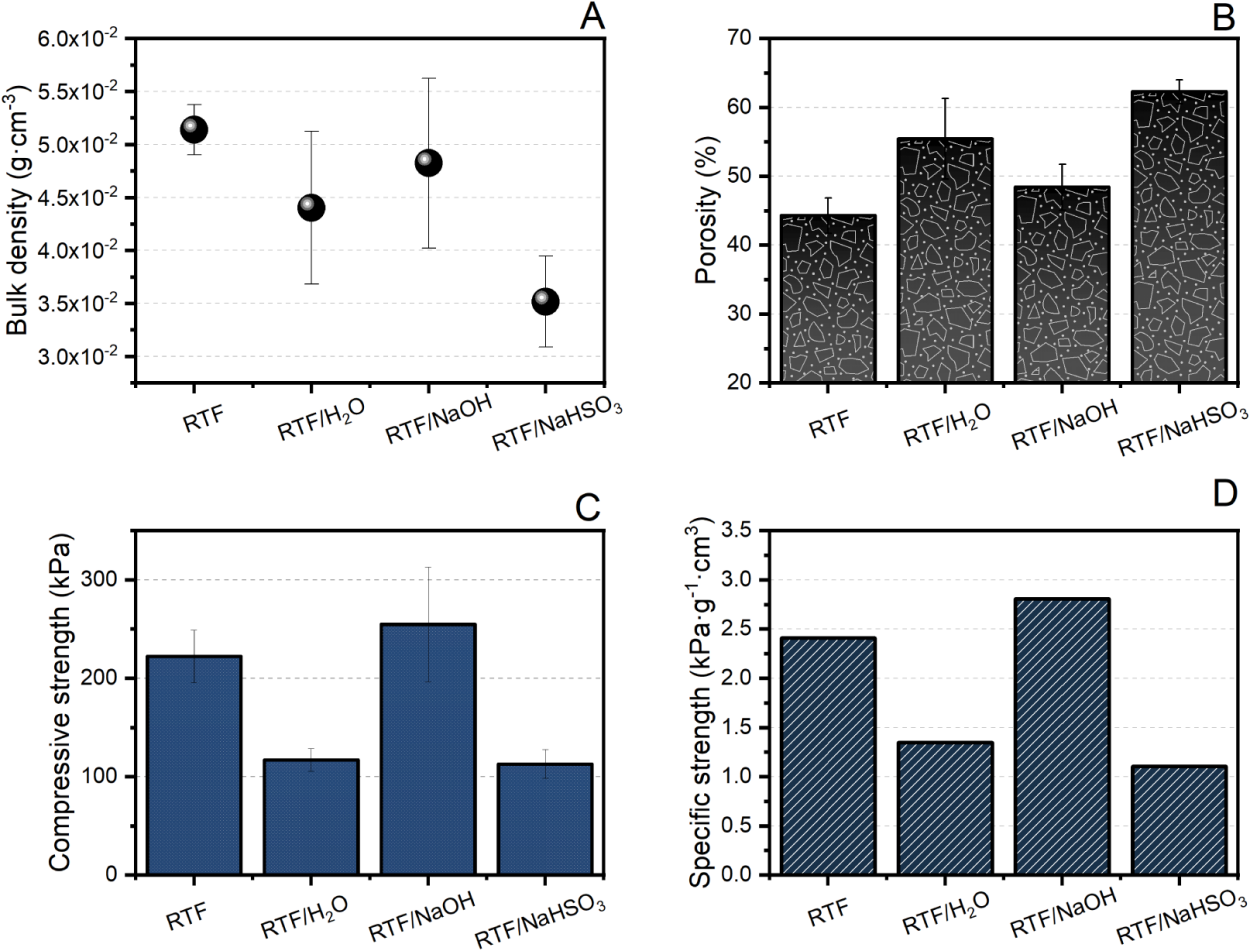
Physical properties of tannin–furfuryl alcohol rigid foams: (A) bulk density, (B) porosity, (C) compressive strength, and (D) specific compressive strength.

The apparent density of the foams (Figure 4A) varied across the formulations, with RTF/NaHSO_3_ exhibiting the lowest density (∼3.5 × 10^–2^ g·cm^–3^), while RTF showed the highest (∼5.0 × 10^–2^ g·cm^–3^). These differences directly reflect the effect of extract addition on the formation of the cellular structure. Rigid tannin–furfuryl alcohol foams are influenced by several factors affecting their density. The addition of polymeric diphenylmethane diisocyanate (pMDI) increases foam density and improves their mechanical performance,^48^ with formulation modifications resulting either in lightweight foams (∼0.050 g·cm^–3^) or in stronger foams with densities up to ∼0.180 g·cm^–3^.^49^ It is worth noting that the pH of the catalytic medium also influences foam formation, with acid- or base-catalyzed foams showing properties comparable to synthetic phenolic foams.^50^ An alternative approach for producing lower-density foams is the incorporation of cellulose nanofibrils (CNFs) as reinforcing agents, eliminating the need for chemical cross-linking and yielding foams that are 30% stronger and 25% lighter than those cross-linked with formaldehyde.^51^

Complementary to density, porosity values (Figure 4B) confirm the inverse trend, indicating that RTF/NaHSO_3_ foams exhibit the highest porosity (∼63%), whereas RTF foams are the least porous (∼46%). The higher porosity may be associated with changes in the viscosity of the reaction medium or with the presence of sulfonated agents in the *T*_NaHSO_3__ extract, which influence bubble formation and stabilization during foam expansion. The RTF/H_2_O and RTF/NaOH foams displayed intermediate porosities (∼55% and ∼49%, respectively), suggesting that, despite the changes in the extracts, the incorporation of aqueous or alkaline additives did not drastically compromise the development of the cellular matrix.

The mechanical performance of the foams was assessed by uniaxial compression testing (Figure 4C). The RTF foam exhibited good compressive strength (∼230 kPa), whereas the addition of the NaOH extract resulted in the best mechanical performance (∼280 kPa). This increase may be attributed to the higher cohesion of the matrix provided by the interaction between the alkaline extract and tannin during cross-linking, possibly favoring the formation of denser cross-links or stiffer internal structures. Reported compressive strengths for tannin–furfuryl alcohol rigid foams range from 0.1 to 1 MPa. Lacoste et al.^52^ investigated the mechanical and physical properties of pine tannin-based foams, reporting values from 0.028 MPa for the least dense foam up to 1.75 MPa for the densest one. In other studies, Sepperer et al.^53^ demonstrated that mechanical agitation combined with surfactant optimization produced foams with compressive strengths of ∼0.8 MPa, while Chen et al.^54^ described self-blowing systems incorporating humins that achieved values above 1 MPa.

Conversely, foams modified with *T*_H_2_O_ and *T*_NaHSO_3__ extracts exhibited the lowest compressive strengths (∼120–140 kPa). The high porosity observed in these formulations likely compromised structural integrity, as more porous foams tend to have thinner cell walls, making them less resistant to deformation. This trend is further confirmed by the specific stress values (Figure 4D), which normalize mechanical resistance by foam density and serve as a more direct indicator of mechanical efficiency. The RTF/NaOH foam achieved the highest specific stress (∼3.0 kPa·g^–1^·cm^3^), followed by RTF (∼2.5 kPa·g^–1^·cm^3^), whereas the *T*_NaHSO_3__ and *T*_H_2_O_ foams showed the lowest values (∼1.4–1.8 kPa·g^–1^·cm^3^).

The analysis of foam cellular structure by cell-length distribution histograms, combined with SEM images, reveals significant morphological differences among the formulations, which help explain the previously discussed physical and mechanical behaviors. In polymeric foams, cell size is strongly influenced by the type and amount of blowing agent employed (e.g., pentane produces larger cells than diethyl ether at comparable densities).^48^ The consistent use of the same blowing agent in this work allows isolating the impact of the tannin extracts.

The RTF foam (Figure 5A,E) exhibits a relatively coherent structure, with a unimodal distribution of cell sizes predominantly between 0 and 150 μm, with an average of 74.94 ± 5.53 μm. This uniformity in cell distribution favors structural organization and is directly associated with the good compressive strength observed (Figure 4C), while also justifying the intermediate density and controlled porosity of this formulation.

**Figure 5:**
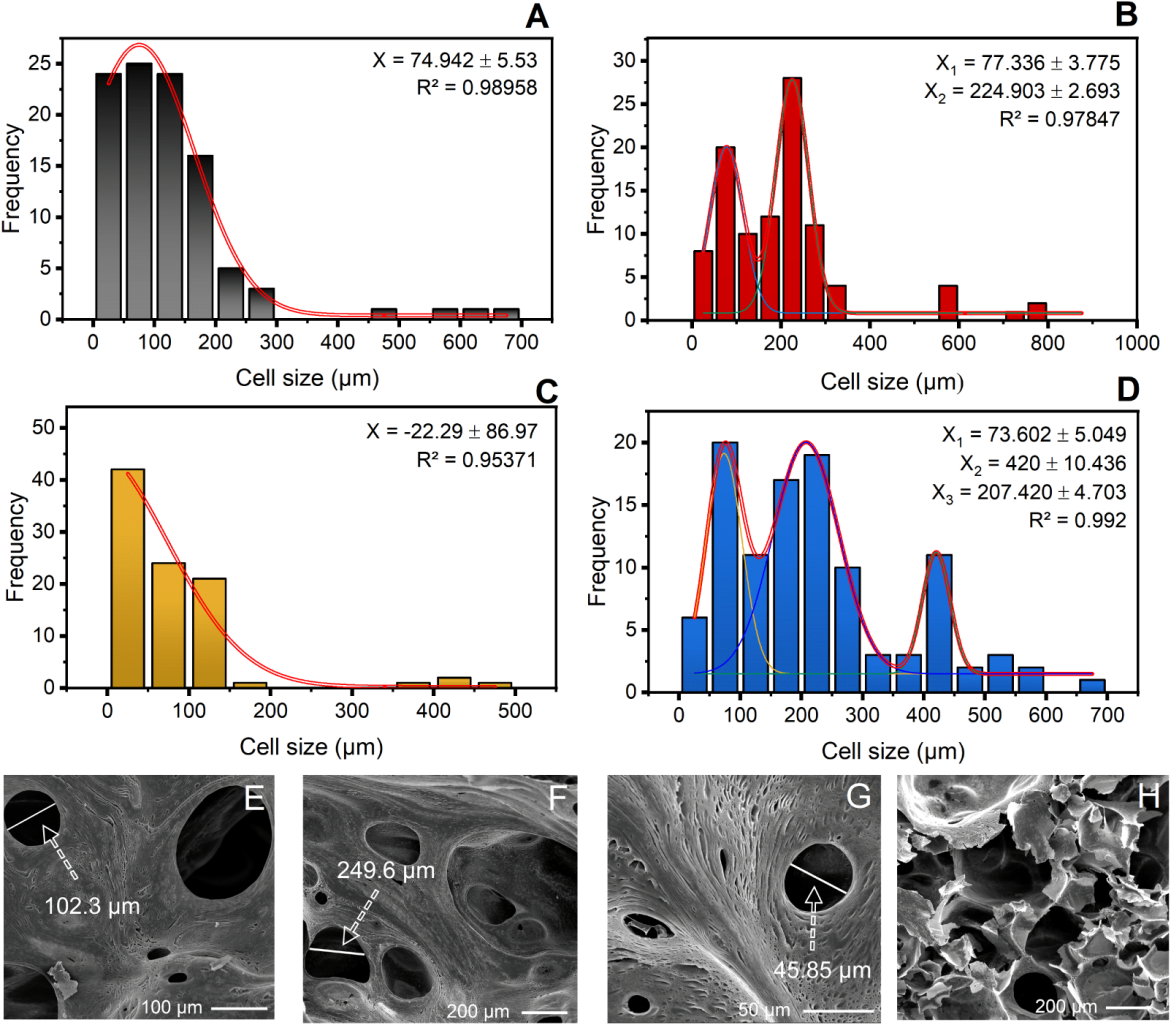
Cell size distribution (A–D) and scanning electron microscopy (SEM) micrographs (E–H) of tannin rigid foams: RTF (A, E), RTF/H_2_O (B, F), RTF/NaOH (C, G), and RTF/NaHSO_3_ (D, H).

The RTF/H_2_O foam (Figure 5B,F), in contrast, displays a bimodal cellular structure, with two distinct peaks at 77.34 ± 3.77 μm and 224.90 ± 2.69 μm. Such morphological heterogeneity may indicate failures in cell nucleation and growth, resulting in less cohesive foam. This is reflected in its lower compressive strength and higher water uptake previously reported, as larger and irregular cells facilitate capillary water transport and compromise structural integrity.

For the RTF/NaOH foam (Figure 5C,G), the fitted histogram yielded an unrealistic negative mean (−22.29 ± 86.97 μm), suggesting that the statistical model did not properly represent the real distribution. Nonetheless, visual inspection of the histogram and SEM images reveals that the highest frequency of cell lengths lies below 50 μm. This behavior is not directly linked to the phenolic content (the lowest among the extracts), but rather to the alkaline nature of the extract. Although viscosity was not directly measured in this study, it is hypothesized that the high ion concentration and alkalinity of the system promote functional group ionization, likely increasing the colloidal viscosity of the matrix during foam formation. Similar phenomena have been reported in tannin-based systems where alkaline conditions modified the polymerization kinetics, leading to viscosity changes that directly impact cell morphology and density.^55^ The viscosity of a polymeric system has a major influence on foam porosity and morphology. Lower viscosities generally lead to smaller cell sizes, more uniform cell distribution, and higher cell population density—but only up to a point at which viscosity can still sustain cell expansion.^56,57^ Increasing viscosity through precuring or filler addition has been shown to improve pore uniformity and overall foam morphology.^58^ However, excessively high viscosity can hinder fluidity and impair foaming quality. In microfluidic foaming, for instance, higher viscosity narrows the range of possible bubble fractions and diameters, limiting the geometrical properties of the resulting materials.^59^ In the case of RTF/NaOH foam, the higher viscosity restricted gas bubble expansion and delayed cell coalescence, resulting in a more compact foam with smaller average cell size, higher relative density, and superior compressive strength, as previously demonstrated. The dense cellular structure also contributes to enhanced thermal performance and fire resistance.

Finally, the RTF/NaHSO_3_ foam (Figure 5D,H) presents the most heterogeneous cellular structure among all formulations. Its histogram displays three distinct peaks (73.60 ± 5.05 μm, 420 ± 10.44 μm, and 207.42 ± 4.70 μm), indicating a highly irregular distribution. SEM images confirm this disorder, showing cells of varying sizes and morphologies. Such a microstructure directly contributes to the high porosity and low density reported earlier. However, the thin and discontinuous cell walls render this foam structurally fragile, leading to the lowest compressive strength of all formulations. The high expansion observed during synthesis suggests that the expansion kinetics likely outpaced the curing rate. This behavior may be associated with the high solubility of the NaHSO_3_ extract and the presence of sulfonate groups, which increase component dispersion and reduce the initial viscosity of the formulation.^60^ As a result, bubbles overgrow before the matrix solidifies, producing a lightweight, highly porous foam with poor mechanical resistance.

Water absorption capacity and moisture content are critical properties for tannin-based materials, particularly in applications involving exposure to humidity or requiring dimensional stability. The results are presented in Figure 6A,B. The absorption curves show that all foams reached equilibrium within the first hours of immersion. The RTF/H_2_O foam exhibited the highest water uptake (∼10%), followed by the control foam (RTF, ∼8%). Foams incorporating *T*_NaOH_ and *T*_NaHSO_3__ extracts displayed the lowest absorptions, stabilizing between 5 and 6%.

**Figure 6:**
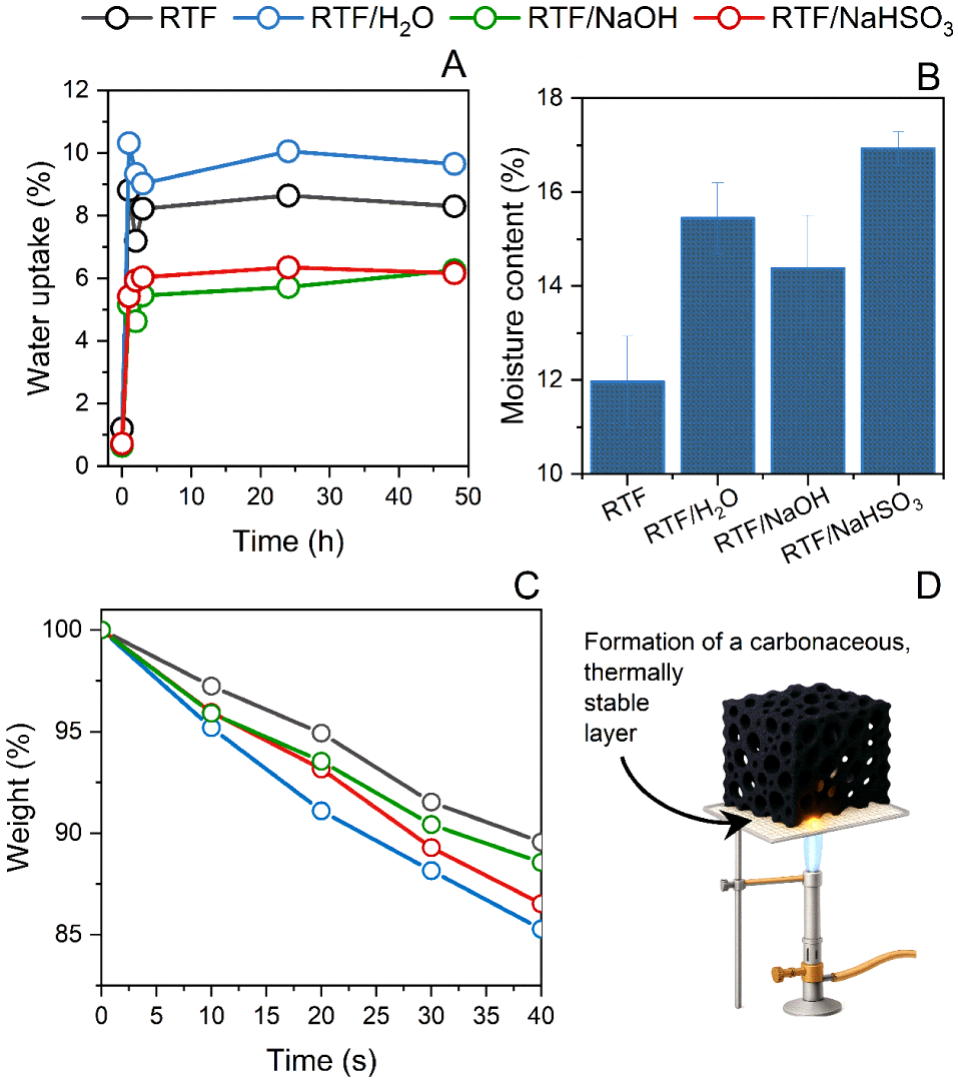
Water interaction behavior (A) water uptake and (B) moisture content), (C) mass loss kinetics of tannin–furfuryl alcohol rigid foams and (D) illustration highlighting char layer formation upon fire exposure.

This behavior is governed by the interplay between the chemical affinity of the matrix and its cellular morphology. As observed in the SEM analysis (Figure 5), the RTF/H_2_O foam presents a heterogeneous structure with larger cells. This morphology typically correlates with a higher proportion of open cells, which, combined with the abundance of hydrophilic hydroxyl groups (confirmed by the broad FTIR band at ∼3300 cm^–1^), facilitates water ingress and retention via capillary action.

Conversely, the RTF/NaOH foam exhibits a more compact structure with smaller cells and thicker walls (consistent with its higher density). This denser network acts as a physical barrier to water diffusion, hindering immediate capillary uptake. For the RTF/NaHSO_3_ foam, despite its high porosity and thin cell walls, the low water absorption suggests that the specific surface chemistry—likely composed of condensed or sulfonated structures with lower polarity—dominates the interaction, reducing the wettability of the solid phase and limiting water penetration into the cellular structure.^20^

Opposite to the trend observed for water absorption, the equilibrium moisture content of RTF/NaOH and RTF/NaHSO_3_ foams was higher than that of the control foam, with RTF/NaHSO_3_ showing the highest value (∼17%). This indicates that, although these formulations absorb less liquid water in the short term, they are more prone to retaining atmospheric moisture at equilibrium. This distinction suggests that water absorption and moisture content are governed by different phenomena: while water absorption reflects the permeability of the structure to liquid water ingress in the short term, moisture content is linked to the affinity of the matrix for water vapor and its internal and surface hygroscopic capacity. The presence of residual ions from the chemical agents (Na^+^, SO_3_^2–^) in the foams may enhance hygroscopicity by favoring water retention through adsorption, even without strongly affecting liquid uptake. Additionally, structural rearrangements and possible porosity changes induced by chemical modification with the extracts may further contribute to these differences.

The fire resistance of the foams was evaluated through the mass loss kinetics under direct flame exposure, with results expressed as the remaining mass over 40 s (Figure 6C). The mass loss curve reflects the thermal stability, flammability, and degradation rate of the modified foam formulations. The RTF foam exhibited the best thermal performance, showing the lowest mass loss over time. After 40 s of flame exposure, RTF retained approximately 89% of its original mass, demonstrating high thermal resistance and self-extinguishing behavior, characteristic of tannin–formaldehyde foams.^51^ In contrast, the RTF/H_2_O foam showed the highest mass loss (∼85%), indicating reduced thermal resistance when aqueous extracts were incorporated into the formulation. The RTF/NaHSO_3_ and RTF/NaOH foams displayed intermediate performance, retaining ∼86% and ∼88% of the initial mass, respectively, suggesting that chemical modification with these extracts has a less severe effect on the material’s thermal stability. The remarkable fire resistance observed (mass retention >85%) places these eucalyptus bark tannin foams among the high-performance bioinsulators, distinguishing them from standard synthetic counterparts. Recent comparative studies by Parcheta-Szwindowska et al.^61^ demonstrated that while rigid polyurethane (PU) foams undergo rapid combustion with mass losses exceeding 90% and release toxic smoke, tannin-based materials naturally exhibit self-extinguishing behavior due to their high aromatic content. Furthermore, our formulation achieved this thermal stability without the need for external flame retardants. This is a significant advantage compared to recent works such as Kim et al.^62^ and Soykan et al.,^63^ where phosphorus-based additives or boron compounds were required to achieve similar char yields in lignin- and soy-based foams. The ability of the produced foams to maintain structural integrity under flame, forming a protective carbonaceous crust, aligns with the “intumescent-like” mechanism for flavonoid-derived matrices, confirming that the extraction method preserved the essential polyphenolic structure required for thermal protection.

The lower thermal resistance of RTF/H_2_O may be associated with the higher incorporation of hydrophilic and thermolabile phenolic compounds, such as flavonoids and simple phenolic acids, abundant in the *T*_H_2_O_ aqueous extract. These compounds can act as combustion propagators or decompose more easily under heat, accelerating the formation of combustible volatiles.^64^ In contrast, *T*_NaOH_ and *T*_NaHSO_3__ extracts likely contain more condensed or partially oxidized phenolic structures (e.g., oligomers or sulfonated acids). These structures tend to form more thermally stable char residues during degradation, despite the lower total phenolic content.^65^ This explains the intermediate and stable performance of RTF/NaOH and RTF/NaHSO_3_ foams under flame exposure.

Another factor to consider is the interaction between thermal resistance and porosity/water absorption. RTF/H_2_O foam exhibited higher water uptake and hydrophilic compound content, which may result in increased permeability and more oxygen-containing groups available for thermal oxidation. More open and hydrophilic foams also tend to retain more oxygen within their pores, promoting combustion and accelerating mass loss. Conversely, foams with less polar extracts, such as RTF/NaOH, may have lower open porosity and fewer reactive oxygen groups, contributing to the formation of a protective char layer during thermal attack, thereby slowing flame propagation (Figure 6D). For comparison purposes, all data of the different foams are displayed in Table 2.

**Table 2:** Physical, Mechanical, and Thermal Properties of the Rigid Tannin–Furfuryl Alcohol Foams Produced with Different Extracts

	RTF	RTF/H_2_O	RTF/NaOH	RTF/NaHSO_3_
Density (10^–2^ gcm^–3^)	5.14 ± 0.24	4.4 ± 0.72	4.8 ± 0.8	3.5 ± 4.3
Porosity (%)	44.28 ± 2.57	55.42 ± 5.85	48.42 ± 3.34	62.25 ± 1.75
Compressive strength (kPa)	222.45 ± 26.52	117.08 ± 11.59	254.8 ± 58.04	112.89 ± 14.66
Water uptake at 24 h (%)	8.64	10.06	5.72	6.35
Moisture content (%)	11.97 ± 0.97	15.44 ± 0.76	14.38 ± 1.13	16.93 ± 0.36
Mass after fire (%)	89.57	85.28	86.53	88.56
*T*_max_ degradation (°C)	460	454.8	458.2	447.5

## 4 Conclusions

This study demonstrates that the selection of the extraction medium is a critical and strategic step for tailoring the final properties of tannin-based foams derived from eucalyptus bark. It was elucidated that a functional trade-off exists between mechanical performance and the preservation of bioactive properties, governed directly by the extraction chemistry. Alkaline extraction with NaOH, despite reducing the total phenolic content, proved to be the optimal route for producing mechanically robust foams with high compressive strength and a dense cellular structure, making them suitable for applications where structural integrity is paramount. In contrast, aqueous extraction preserved the antioxidant-rich phenolic fractions but yielded a more hydrophilic and mechanically weaker foam matrix, indicating its potential for different applications where specific chemical reactivity is prioritized overload-bearing capacity. Furthermore, the use of sodium bisulfite resulted in lightweight, highly porous foams whose structural integrity was compromised by a highly heterogeneous cellular network, confirming that each medium imparts a unique morphological signature to the final material. Ultimately, this work transcends a simple characterization of extracts by establishing a clear link between the extraction process and material performance. It provides a foundational understanding for the rational design of biobased foams with predictable and tunable properties, reinforcing the potential of forestry residues as valuable feedstocks for advanced sustainable materials. While this study established the correlation between extraction media and foam performance, future studies focused on the in situ rheological monitoring of the foaming process are recommended. Such investigations would help to fully elucidate the kinetic mechanisms governing cell nucleation and growth in foams modified with alkaline and saline extracts.

## Data Availability

The study’s data was presented in tables and figures. If desired, data can be obtained by contacting the corresponding author through a reasonable request.
